# Theonellamide G, a Potent Antifungal and Cytotoxic Bicyclic Glycopeptide from the Red Sea Marine Sponge *Theonella swinhoei*

**DOI:** 10.3390/md12041911

**Published:** 2014-04-01

**Authors:** Diaa T. A. Youssef, Lamiaa A. Shaala, Gamal A. Mohamed, Jihan M. Badr, Faida H. Bamanie, Sabrin R. M. Ibrahim

**Affiliations:** 1 Department of Natural Products, Faculty of Pharmacy, King Abdulaziz University, Jeddah 21589, Kingdom of Saudi Arabia; E-Mails: gahussein@kau.edu.sa (G.A.M.); jibrahim@kau.edu.sa (J.M.B.); 2 Natural Products Unit, King Fahd Medical Research Center, King Abdulaziz University, Jeddah 21589, Kingdom of Saudi Arabia; E-Mail: lshalla@kau.edu.sa; 3 Suez Canal University Hospital, Suez Canal University, Ismailia 41522, Egypt; 4 Department of Pharmacognosy, Faculty of Pharmacy, Al-Azhar University, Assiut Branch, Assiut 71524, Egypt; 5 Department of Pharmacognosy, Faculty of Pharmacy, Suez Canal University, Ismailia 41522, Egypt; 6 Department of Clinical Biochemistry, Faculty of Medicine, King Abdulaziz University, Jeddah 21589, Kingdom of Saudi Arabia; E-Mail: fbamanea@kau.edu.sa; 7 Department of Pharmacognosy, Faculty of Pharmacy, Assiut University, Assiut 71526, Egypt; E-Mail: sribrahim@taibahu.edu.sa; 8 Department of Pharmacognosy and Medicinal Chemistry, Faculty of Pharmacy, Taibah University, Al Madinah Al Munawwarah 41477, Kingdom of Saudi Arabia

**Keywords:** *Theonella swinhoei*, glycopeptide, theonellamide G, antifungal activity, cytotoxic activity

## Abstract

In our search for bioactive metabolites from marine organisms, we have investigated the polar fraction of the organic extract of the Red Sea sponge *Theonella swinhoei*. Successive chromatographic separations and final HPLC purification of the potent antifungal fraction afforded a new bicyclic glycopeptide, theonellamide G (**1**). The structure of the peptide was determined using extensive 1D and 2D NMR and high-resolution mass spectral determinations. The absolute configuration of theonellamide G was determined by chemical degradation and 2D NMR spectroscopy. Theonellamide G showed potent antifungal activity towards wild and amphotericin B-resistant strains of *Candida albicans* with IC_50_ of 4.49 and 2.0 μM, respectively. Additionally, it displayed cytotoxic activity against the human colon adenocarcinoma cell line (HCT-16) with IC_50_ of 6.0 μM. These findings provide further insight into the chemical diversity and biological activities of this class of compounds.

## 1. Introduction

The order Lithistida include the genera *Theonella*, *Discodermia*, *Aciculites*, *Microscleroderma*, and *Callipelta*. Lithistid sponges have been shown to yield a wide variety of bioactive marine natural products that include unique cyclic peptides and depsipeptides [1,2,3]. The genus *Theonella* is known to be a rich source of structurally diverse, biologically active peptides [1] including polytheonamides [4], cyclotheonamides [5], theonellapeptolides [6], theonellamides [7], theonegramides [8], keramamides [9], mozamides [3], mutoporins [10], microsclerodermins [11], cupolamide [12], oriamide [13], and cyclolithistide A [14]. Many *Theonella* derived peptides demonstrate potent cytotoxicity [4,7,12,13], thrombin inhibition [5], phosphatase inhibition [10], protease inhibition [5], antifungal [7,8,11,14,15],and anti-HIV properties [16]. Our previous investigation on the Red Sea *Theonella swinhoei* led to the isolation of several macrolides including swinholide A, I and hurghadolide A [17]. As a continuation of this work, we have investigated the polar active fraction of the organic extract of the sponge. Here, we describe the isolation, structure elucidation, and biological activity of a new bicyclic glycopeptide, theonellamide G (**1**). Theonellamides A–F were previously isolated from *Theonella* sp. [7,18]. Theonellamides A–E have been found to possess cytotoxic activity, while a potent antifungal activity was reported for theonellamide F [7,18]. Theonellamides represent a new class of sterol-binding molecules that induce glucan overproduction, damage cellular membranes, and activate Rho1-mediated 1,3-β-d-glucan synthesis [19,20,21]. The absolute stereochemistry of the amino acid residues of theonellamides was determined using chemical methods, chiral GC, and Marfey’s analyses. Interestingly, the Red Sea sample did not contain any of the previously reported theonellamides.

## 2. Results and Discussion

### 2.1. Purification of Compound 1

**Figure 1:**
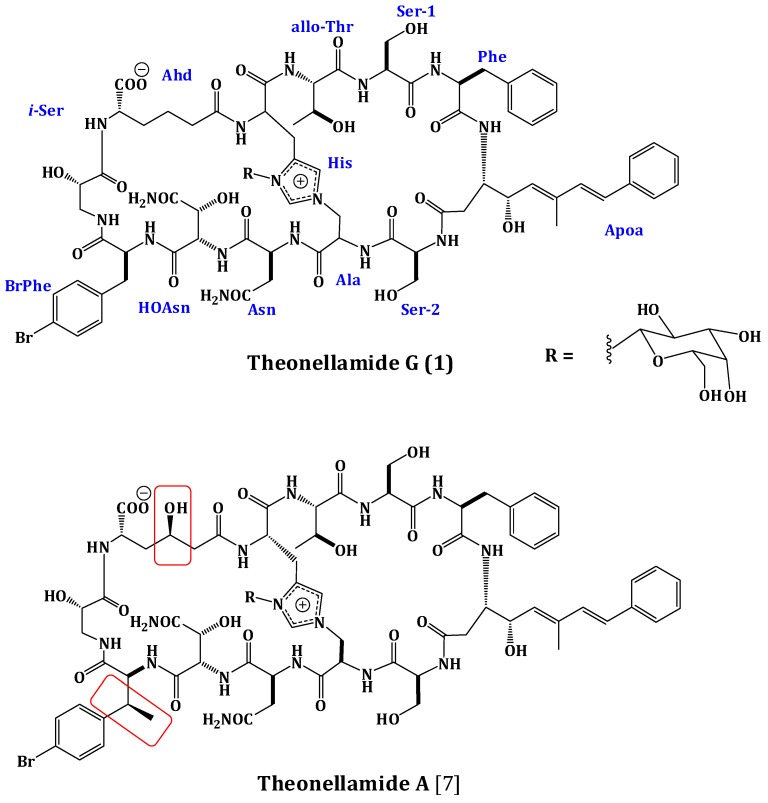
Structures of Theonellamide G (**1**) and Theonellamide A.

The frozen sponge was extracted with a mixture of MeOH/CH_2_Cl_2_ (1:1). The combined extracts were suspended in MeOH/H_2_O (9:1) and partitioned between *n*-hexane and 90% MeOH followed by fractionation between CH_2_Cl_2_ and 60% MeOH. The CH_2_Cl_2_ fraction was subjected to size exclusion chromatography on Sephadex LH-20 (Merck, Darmstadt, Germany) followed by ODS flash column chromatography (Yamazen Corporation, Osaka, Japan) of the active antifungal fraction. Final HPLC purification of the polar and potent antifungal fraction on a preparative C30 HPLC column afforded theonellamide G (**1)** (Figure 1).

### 2.2. Structure Elucidation of Compound 1

**Figure 2:**
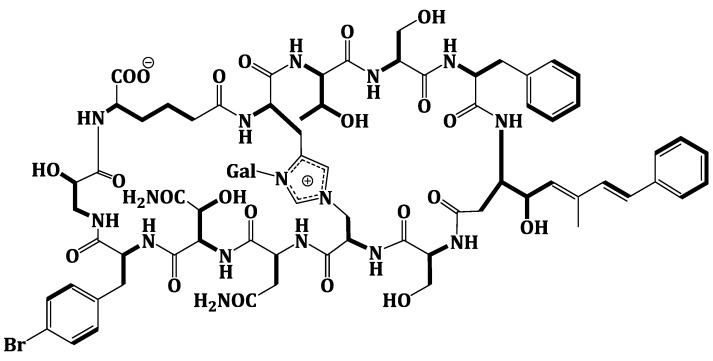
Observed ^1^H-^1^H COSY Correlations of Theonellamide G (**1**).

Compound **1** was obtained as an optically active powder. The molecular formula of **1** was C_75_H_97_BrN_16_O_27_ on the basis of HRFABMS and NMR data, requiring 36 degrees of unsaturation. Compound **1** is 30 mass units less than theonellamide A (Figure 1) [7], indicating loss of methyl and hydroxyl functionalities. The UV absorption bands at 289 and 306 nm suggested the presence of (5*E*,7*E*)-3-amino-4-hydroxy-6-methyl-8-phenyl-5,7-octadienoic acid (Apoa) moiety in **1** [7,15,18].The IR spectrum showed absorption bands at 3324 and 1655 cm^−1^, corresponding to amino and carbonyl groups, respectively. The NMR data of **1** were similar to those of theonellamide A but new signals for *p*-bromophenylalanine residue in **1**, replacing the signals of the β-methyl-*p*-bromophenylalanine (β-MeBrPhe) residue in theonellamide A, were observed [7,15,18] (Supplementary Figures S1–S6). The signals at δ_H_ 4.34 (1H, m, 9-αH)/55.8, 3.01 (1H, brd, *J* = 14.4 Hz, 9-βHa) and 2.65 (1H, m, 9-βHb)/δ_C_ 37.2, 7.21 (2H, d, *J* = 6.6 Hz, H-2‴, 6‴)/δ_C_ 129.2, 7.28 (2H, d, *J* = 6.6 Hz, H-3‴, 5‴)/δ_C_ 131.8, 120.6 (C-4‴), and 172.6 (9-CO) (Table 1) were consistent with the *p*-bromophenylalanine residue [7,15,18]. The ^1^H-^1^H COSY correlations from 9-αH to 9-βHa and 9-βHb_,_ H-2‴ to H-3‴, and H-5‴ to H-6‴, as well as, the HMBC cross peaks of 9-αH to 9-CO, 9-βH to 9-CO, C-1‴, and C-2‴, H-2‴ and H-6‴ to C-1‴, C-4‴, and H-3‴ and H-5‴ to C-1‴, C-2‴, and C-6‴, supported the assignment of the *p*-bromophenylalanine (BrPhe) residue. Furthermore, the chemical shift of C-1‴ in **1** (δ_C_ 137.7) compared to 141.6 ppm in theonellamide A [7] supported the absence of the β-methyl group in *p*-bromophenylalanine moiety in **1**. In addition, a new spin system consisting of three coupled methylenes at δ_H_ 2.22 and 2.01 (11-αH), 1.37 and 1.02 (11-βH), and 1.78 and 1.53 (11-γH) together with a methine at δ_H_ 4.57 (11-δH) and NH group at δ_H_ 7.63 (11-NH) was observed in ^1^H-^1^H COSY spectrum,suggesting the presence of2-aminohexanedioic acid (Ahd) residue (Figure 2). The HMBC cross peaks from 11-αH to 11-CO and 11-βC, 11-γH to 11-αC, and 11-δH to 11-βC and 11-COO^−^ corroborated this spin system.

**Figure 3:**
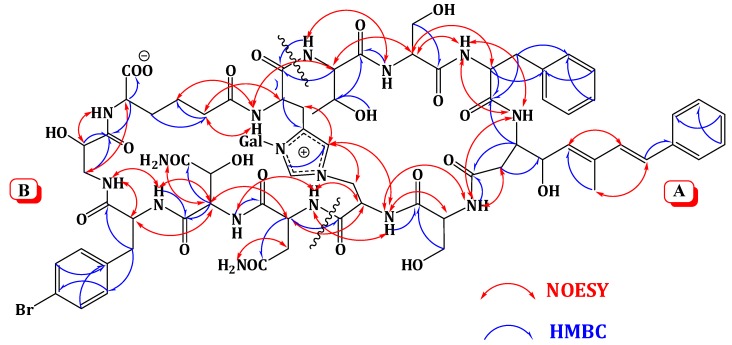
Significant Observed NOESY and HMBC Correlations of Theonellamide G (**1**).

Extensive analysis of the 1D and 2D NMR data (Supplementary Figures S1–S6). of **1** revealed the presence of 12 spin systems which could be assigned to 12 amino acid residues [Thr, two Ser residues, Ala, Asn, OHAsn, Phe, BrPhe, His, *i*-Ser, Apoa, and Ahd] (Figure 2 and Figure 3). They were confirmed by the presence of 12 carbonyls between 170.0 and 175.0 ppm and 12 α-carbons in the region of 36.9–70.2. The carbonyl carbon atδ_C_ 175.0 was assigned to the α-free carboxylic acid group of Ahd [7,15,18]. In addition, the ^1^H and ^13^C NMR spectra showed signals at δ_H_/δ_C_ 5.03/89.0, 3.83/69.5, 3.45/73.7, 3.66/69.8, 3.67/79.0, and 3.76 and 3.36/62.0, indicating the presence of a hexose moiety. It was confirmed by the observed ^1^H-^1^H COSY and HMBC correlations (Table 1). It was identified as d-galactose based on the ^1^H and ^13^C NMR data in addition to co-TLC with authentic sample upon the acid hydrolysis of **1** using solvent system S2 (R*_f_* = 0.33) [7,15,18]. The ^1^H and ^13^C NMR chemical shift values of the galactose moiety were similar to those reported in theonellamides A and E [7] (Supplementary Figures S1 and S2). Thecoupling constant value (*J*_H-1, H-2_ = 9.0 Hz) indicated an axial configuration of the anomeric proton [7,15,18]. Thus, 34 of the 36 double bond equivalent required by the molecular formula were encountered by the amino acid residues and galactose moiety, indicating **1** was a bicyclic peptide. The sequence of amino acids and the bicyclic nature of **1** were established by a detailed examination of the NOESY and HMBC spectra (Figure 3; Supplementary Figures S5 and S6). In the NOESY spectrum, the cross peaks observed between the α-H and NH group of adjacent amino acids and between the NH and NH of adjacent residue established the presence of two substructures; *allo*Thr-Ser-Phe-Apoa-Ser-Ala (substructure A) and Asn-OHAsn-BrPhe-*i*Ser-Ahd-His (substructure B). In particular, the two substructures were corroborated by HMBC (^2^*J*_CH_ and ^3^*J*_CH_) correlations of NH and α-H of each amino acid to the amide carbonyl carbons. The sequence *allo*Thr-Ser-Phe-Apoa-Ser-Ala was confirmed by the key HMBC cross peaks of 1-NH/2-CO, 3-NH/2-CO, 4-βH/3-CO, 5-NH/4-CO, and 6-NH/5-CO. The HMBC correlations of 8-NH/7-CO, 9-NH/8-CO, 10-NH/9-CO, 11NH/10-CO, and 12NH/11-CO proved the substructure B. Substructures A and B were connected on the basis of NOESY correlations from 1-αH to 12-NH, 1-NH to 12-αH, 6-αH and 6-βH to 7-αH and 7-NH, 6-NH to 7-NH, H-2″″ to 6-βH and 6-NH, H-5″″ to 6-αH and 6-βH, and further confirmed by the HMBC correlations of 1-αH to 12-CO, 6-βH to C-2″″, and 7-αH and 7-NH to 6-CO.

The attachment of the galactose moiety at postion-3 of His moiety was apparent from the NOESY correlation of the anomeric proton at δ_H_ 5.03 to H-2″″ and secured by its HMBC cross peaks to C-2″″ and C-4″″. The absolute configuration of **1** was determined by acid hydrolysis followed by chiral GC-MS and Marfey’s analyses. Chiral GC-MS of the acid hydrolysate and LC-MS of the hydrolysate product of **1** derivatized with *N*-α-(5-fluoro-2,4-dinitrophenyl)-l-leucinamide (Marfey’s reagent) indicated the presence of l-Asn, l-*allo*Thr, l-Ser, 2*S*-*i*-Ser, l-Phe, l-BrPhe, 2*S*,3*R-*HOAsn, and d-galactose. However, the absolute stereochemistry of Apoa, Hisala, and Ahd residues could not be determined. This stereochemical assignment was confirmed by comparison of NMR coupling constant values and chemical shifts with literature [7,15,18]. The *E*,*E* geometry of the olefinic double bonds Δ^4δ^ and Δ^4ζ^ of Apoa was assigned on the basis of the intense NOESY cross peaks between δ_H_ 5.12 (1H, m, 4-δH) and 6.47 (1H, d, *J* = 16.2 Hz, 4-ζH) and between δ_H_ 6.56 (1H, d, *J* = 16.2 Hz, 4-ηH) and 1.63 (3H, s, 4-εCH_3_) and confirmed by the coupling constant values and the ^13^C chemical shift of 4-εCH_3_ (δ_C_ 13.4). In conclusion, comparison of the spectral data of **1** with those of theonellamide A suggested the replacement of the β-MeBrPhe and α-amino-γ-hydroxyadipic acid in theonellamide A with BrPhe and Ahd in **1**. Thus, the structure of **1** was unambiguously elucidated as depicted and the trivial name theonellamide G was given to it.

Theonellamide G (**1**) showed potent antifungal activity towards wild and amphotericin B-resistant strains of *Candida albicans* with IC_50_ of 4.49 and 2.0 μM, respectively, compared to 1.48 μM for the positive antifungal control amphotericin-B against the wild type (Table 2). Additionally, compound **1** displayed cytotoxic activity against the human colon adenocarcinoma cell line (HCT-16) with IC_50_ of 6.0 μM, compared to 2.0 μM for etoposide (positive anticancer control) (Table 2). 

**Table 1:** NMR Spectroscopic Data of Theonellamide G (**1**) (DMSO-*d*_6_:H_2_O, 4:1).

Amino acid	C	δ_H_ m (*J* in Hz)	δ_C _m	HMBC	NOESY
**allo-Thr^1^**	CO	−	173.1 C	−	−
	α	4.18 d (9.8)	58.9 CH	1 β, 12CO	1γ, 2α, 12NH
	β	3.55 m	69.0 CH	1CO	12α, 2NH
	γ	0.84 brs	21.4 CH_3_	1α, 1β	1α, 2NH, 12α, 12β
	NH	7.65 d (7.8)	−	12CO	2α, 2NH, 12α, 5″″
	OH	5.12 m	−	1β	−
**Ser-1^2^**	CO	−	170.0 C	−	−
	α	4.45 m	56.6 CH	−	3α, 3NH
	β	3.64 m	61.1 CH_2_	2α, 2CO	2α, 3NH
	NH	7.73 d (3.6)	−	1CO	1NH, 3α, 3NH
**Phe^3^**	CO	−	171.6 C	−	−
	α	4.55 t (8.3)	54.9 CH	3CO, 1′	2α, 2NH
	β	2.81 dd (13.3, 6.8) 2.67 m	39.3 CH_2_	3α, 3CO, 1′, 2′, 3′	2NH
	1′	−	137.2 C	−	
	2′, 6′	7.12 d (6.6)	129.9 CH	1′, 3′, 5′	
	3′, 5′	7.01 t (6.6)	131.8 CH	2′, 6′	
	4″	7.28 d (6.6)	129.7 CH	2′, 6′	
	NH	7.93 d (7.8)	−	2CO	2α, 2NH, 4NH
**Apoa^4^**	CO	−	172.6 C	−	
	α	2.55 q (10.3) 2.30 brd (13.8)	36.9 CH_2_	4γ, 4CO	5NH
	β	4.46 m	52.2 CH	3CO, 4CO	4α, 4γ, 5NH
	γ	4.42 t (8.4)	68.8 CH		5NH, 4β, ε-CH_3_
	δ	5.12 m	132.4 CH	4ζ, 4ε	4β, 4ζ, 4γ
	ε	−	137.9 C		−
	ζ	6.47 d (16.2)	133.9 CH	4δ, 4ε, 1″	4δ
	η	6.56 d (16.2)	128.7 CH	4δ, 4ε, 3ζ, 4ε-CH_3_, 1″	ε-CH_3_
	1″	−	137.2 C	−	−
	2″, 6″	7.12 d (6.6)	129.9 CH	1″, 3″, 5″	
	3″, 5″	7.01 t (6.6)	131.8 CH	2″, 6″	
	4″	7.28 d (6.6)	129.7 CH	2″, 6″	
	ε-CH_3_	1.63 s	13.4 CH_3_	4δ, 4ε, 4ζ	4η, 4γ
	NH	8.45 brs	−	4CO	3NH, 3α, 5NH
**Ser-2^5^**	CO	−	172.8 C	−	−
	α	3.74 m	56.8 CH		4α, 4β, 4NH, 6α, 6NH
	β	3.76 m 3.63 m	62.0 CH_2_	5CO	4α, 4β, 4NH, 6α, 6NH
	NH	7.78 brs	−	4CO	4α, 4NH, 6NH
**Ala^6^**	CO	−	170.0 C	−	−
	α	5.08 m	51.4 CH	6CO	7α, 7NH, 2″″
	β	4.90 brd (12.6)	50.6 CH_2_	2″″	7α, 7NH, 2″″, 5″″
	NH	8.27 d (9.6)	−	5CO	5α, 7NH, 5″″
**Asn^7^**	CO	−	171.4 C	−	−
	α	4.11 t (7.2)	52.9 CH	6CO, 7-CONH_2_	6α, 8α, 6NH
	β	2.36 dt (13.3, 7.2) 2.12 brd (13.3)	37.3 CH_2_	7α, 7-CONH_2_	6NH
	CONH_2_	−	172.7 C	−	−
	NH	7.67 d (11.2)	−	6CO	6α, 6NH
	NH_2_	7.69 brs	−	−	7α, 7β
**HOAsn^8^**	CO	−	170.9 C	−	−
	α	5.34 t (8.4)	54.9 CH	8CO, 8-CONH_2_	7α, 9α, 9β, 9NH
	β	4.22 d (11.7)	72.9 CH	−	−
	CONH_2_	−	174.8 C	−	−
	NH	8.32 brs	−	7CO	7NH
	NH_2_	7.78 s	−	−	8α, 8NH
	OH	6.78 brs	−	−	−
**BrPhe^9^**	CO	−	172.6 C	−	−
	α	4.34 m	55.8 CH	9CO	10NH
	β	3.01 brd (14.4) 2.65 m	37.2 CH_2_	9CO, 9α, 1‴, 2‴	10NH
	1‴	−	137.7 C	−	−
	2‴, 6‴	7.21 d (6.6)	129.2 CH	1‴, 4‴	−
	3‴, 5‴	7.28 d (6. 6)	131.8 CH	1‴, 2‴, 6‴	−
	4‴	−	120.6 C	−	−
	NH	8.71 brs	−	8CO	8α, 10NH
***i*-Ser^10^**	CO	−	171.9 C	−	−
	α	4.17 d (11.2)	70.2 CH	10CO	11NH
	β	3.95 m 2.96 brd (7.2)	43.8 CH_2_	10CO	11δ, 11NH
	NH	7.47 d (7.2)	−	9CO	9α, 9β, 9NH, 11γ, 11NH
**Ahd^−11^**	CO	−	173.1 C	−	−
	α	2.22 m 2.01 m	35.9 CH_2_	11CO, 11β	12α, 12NH
	β	1.37 m 1.02 m	22.7 CH_2_	11γ	12NH
	γ	1.78 m 1.53 m	32.5 CH_2_	11α	12NH
	δ	4.57 t (7.3)	54.9 CH	11β, 11γ, 10CO, 11-COO^−^	10NH, 12NH
	COO^−^	−	175.0 C *		−
	NH	7.63 d (6.6)	−	10CO	10NH, 12NH
**His^12^**	CO	−	171.1 C	−	−
	α	4.82 m	54.5 CH	12CO, 4″″	1γ, 1NH, 11α
	β	3.24 t (13.5) 3.01 brd (14.4)	26.3 CH_2_	12α, 4″″	1NH, 1CO, 11α, 2″″, 5″″
	2″″	8.84 s	137.4 CH	4″″, 5″″	1-Gal, 6β, 6NH, 11β
	4″″	−	131.8 C	−	−
	5″″	7.26 brs	124.4 CH	4″″	6α, 6β, 11β, 12β
	NH	8.40 brs	−	11CO	1NH, 11α, 11β, 5″″
**Gal^13^**	1	5.03 d (9.0)	89.0 CH	2, 3, 2″″, 4″″	12NH, 12β, 2″″
	2	3.83 m	69.5 CH	4, 3	
	3	3.45 m	73.7 CH	4, 5	
	4	3.66 m	69.8 CH	3, 6	
	5	3.67 m	79.0 CH	3, 4	
	6	3.76 m 3.63 m	62.0 CH_2_	4, 5	

* δ value was abstracted from HMBC spectrum.

**Table 2:** Antifungal and Cytotoxic Activities of Theonellamide G (**1**) *^a^*.

Compound	*C. albicans* (W.T.) *^b^*	*C. albicans* (AmBR) *^c^*	HCT-116
	MIC (μM)	MIC (μM)	IC_50_ (μM)
Theonellamide G (1)	4.49	2.0	6.0
Amphotericin B *^d^*	1.48	−	
Etoposide *^e^*	−	−	2.0

*^a^* Upper limit on the antifungal assay is 500 μg/mL; *^b^* Wild type (ATCC 32354); *^c^* Amphotericin B-resistant type (ATCC 90873); *^d^* Positive antifungal control; *^e^* Positive cytotoxic control.

## 3. Experimental Section

### 3.1. General Experimental Procedures

Optical rotation was measured on a JASCO DIP-370 digital polarimeter (Jasco Co., Tokyo, Japan) at 25 °C at the sodium D line (589 nm). UV spectrum was recorded on a Hitachi 300 spectrometer (Hitachi High-Technologies Corporation, Kyoto, Japan). The IR spectrum was measured on a Shimadzu Infrared-400 spectrophotometer (Shimadzu, Kyoto, Japan). NMR spectra were determined on BRUKER Unity INOVA 600 instruments (600 MHz for ^1^H and 150 MHz for ^13^C NMR) (Bruker BioSpin, Billerica, MA, USA). Positive HRFABMS spectrum was determined on a Finnigan MAT-312 spectrometer (ThermoFinnigan GmbH, Tokyo, Japan). HPLC purification was performed on a preparative RP C30 column (Develocil, C30-UG-5, 250 × 20 mm, Phenomenex) (Nomura Chemical, Setouchi-shi, Japan) using 25% *n*-propanol in water. Column chromatographic separation was carried out on Sephadex LH-20 (0.25–0.1 mm, Merck, Darmstadt, Germanyand ODS flash column chromatography (Yamazen Corporation, Osaka, Japan), while reversed phase chromatography was performed on YMC*Gel (ODS-AQ-HG, YMC Europe GmbH, Dinslaken, Germany). TLC analyses were conducted on pre-coated silica gel F_254_ aluminum sheets (layer thickness 0.2 mm, Merck, Darmstadt, Germany). Standard amino acids were purchased from Sigma-Aldrich Chemical Co. (Taufkirchen, Germany) and Trademax Pharmaceuticals & Chemicals Co., Ltd. (Shanghai, China). The solvent systems were used for TLC analyses; CHCl_3_:MeOH (9:1, S1) and CHCl_3_:EtOAc:MeOH:H_2_O (2.8:3.2:3.5:0.5, S2).

### 3.2. Animal Materials

The marine sponge was collected by scuba diving at a depth of 4–5 m of Hurghada in the Red Sea coast. The sponge is cylindrical in shape and dark red-brown in color. The cut-off fragment measures 7.5 cm high and 4.5 cm in diameter. It has a central canal of 1.5 cm diameter leading to a narrow vent with sphincter-like membrane at the top. The *in*-*situ* photo shows the vent to be similar in diameter as the central canal. The surface is slightly bumpy, generally smooth, but furrowed lengthwise. The ectosomal skeleton consists of a dense mass of curved acanthomicrorhabds of 15–24 × 2–3 μm, overlying a lose reticulation of reduced phyllotriaenes with cladome spanning 120–180 μm and thin undivided cladi 55–120 × 4–7 μm in size. A subectosomal region measuring about 1 mm in thickness bridges an area devoid of desmas, the skeleton of which consists of bundles of strongylotes, measuring 25–70 μm in diameter, enclosing 4–20 strongylotes. The latter are slightly anisotylote with either end more or less swollen, 405–620 × 3–6 μm in size. The choanosomal skeleton consists of a loose reticulation of tetraclone desmas strengthened by bundles of strongylotes. Desmas cladomes measure 400–550 μm, rhabds smooth, 120–230 × 15–20 μm, and cladi smooth with simple zygoses, 150–250 × 12–16 μm. Compared with the type specimen there are some differences (lighter skeletal, smooth instead of tuberculated desmas, shorter strongylotes) which are judged to be infraspecific variation. The voucher fragment is registered in the collection of the Zoological Museum of Amsterdam under registration number POR 16637 and in the Red Sea Invertebrates Collection at Faculty of Pharmacy, Suez Canal University, Ismailia, Egypt, under registration number DY-RS-59. 

### 3.3. Extraction and Purifications of Compound 1

The frozen sponge materials (1.5 kg, wet weight) were extracted with a mixture of MeOH/CH_2_Cl_2_ (1:1) (3 × 1000 mL) at room temperature. The combined extracts were concentrated under reduced pressure and suspended in MeOH/H_2_O (9:1) (1000 mL). The resulting mixture was extracted with *n*-hexane (3 × 400 mL) to give 7.2 g of *n*-hexane residue. The remaining methanolic layer was diluted with H_2_O to (3:2) MeOH/H_2_O and then extracted with CH_2_Cl_2_ (3 × 400 mL) to give 2.4 g of CH_2_Cl_2_ residue. The CH_2_Cl_2_ residue was subjected to a Sephadex LH-20 column (Merck, Darmstadt, Germany) using MeOH as an eluent to afford nine fractions. Fraction 4 (730 mg) was subjected to ODS flash chromatography starting with 30% aqueous MeOH through pure MeOH to afford 10 subfractions. The potent antifungal subfraction eluted with 40% H_2_O in MeOH (subfraction 4) (86 mg) was subjected to final HPLC purification on a preparative C30 column (Develocil, C30-UG-5, 250 × 20 mm, Nomura Chemical, Setouchi-shi, Japan) using 25% *n*-propanol in water at a flow rate of 5.5 mL/min to afford compound **1** (11.5 mg).

Theonellamide G (**1**): White amorphous powder; 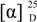 +15.85° (*C* 0.42, MeOH:H_2_O, 4:1); UV (λ*_max_*, MeOH) (log ε) 289 (4.82), 306 (3.57) nm; IR ν_max_ (KBr) 3324, 2965, 1655, 1062 cm^−1^; NMR data, see Table 1; HRFABMS *m*/*z* 1733.5983 (calcd for C_75_H_98_^79^BrN_16_O_27_, 1733.5970, [M + H]^+^). 

### 3.4. Acid Hydrolysis and Absolute Configuration of Amino Acids Using LC-MS Analysis of the Marfey Derivatives of 1

Compound **1** (1.0 mg) was treated with 2 mL 6 N HCl (pa) and heated in sealed ampoule at 110 °C for 24 h under N_2_ gas. The resulting solution was concentrated, with consecutive addition of H_2_O (5 mL) to ensure complete elimination of HCl. To 50 μL of acid hydrolysate (or authentic amino acid standard at comparable concentration), 100 μL FDNPL (1% *N*-(5-flouro-2,4-dinitrophenyl)-l-leucinamide in acetone) and 20 μL 1 M NaHCO_3_ were added. The mixture was heated at 40 °C for 1 h over a hot plate with frequent mixing. After cooling, 10 μL of 2 M HCl was added and then concentrated to dryness before dissolving in 1000 μL MeOH. Standards of l and d amino acids were treated separately with FDNPL in the same manner. The FDNPL derivatives were analyzed using LC-MS by comparison of the retention time and molecular weight with those of standard amino acids FDNPL derivatives [22,23].

### 3.5. Chiral GC-MS Analysis of 1

About 0.2 mg of **1** was placed in sealed ampoule containing 6 N HCl (0.5 mL) and heated at 110 °C for 12 h. After evaporation of the solvent under a stream of N_2_ gas, the residue was dissolved in 10% HC1/MeOH and heated at 100 °C for 30 min. The product was evaporated, dissolved in trifluoroacetic anhydride (50 μL) and CH_2_C1_2_ (50 μL), reacted at 100 °C for 10 min, and evaporated in a stream of N_2_ gas. The residue was dissolved in EtOAc (100 μL). Aliquot (30 μL) was injected into a Hewlett-Packard 5890 GC-MS (Hewlett-Packard, Cary, NC, USA) fitted with an Alltech Chirasil-l-Val capillary column (Varian, Palo Alto, CA, USA). The temperature was ramped form 60 °C to 210 °C over a period of 45 min. The retention time (*t*_R_, min) of the residues in the hydrolysate of **1** matched standards for l-*allo*Thr (14.02; d-*allo*Thr, 12.785), l-Ser (13.42; d-Ser, 12.365), l-Asn (15.78; d-Asn, 15.34), l-Phe (22.967; d-Phe, 22.124), l-BrPhe (32.104; d-BrPhe, 31.68), (2*S*)-*i*Ser (16.344; (2*R*)-*i*Ser, 16.152), and d-Gal (18.582; l-Gal, 18.982). 

### 3.6. Evaluation of Cytotoxic Activity

Cytotoxicity was tested against human colon adenocarcinoma (HCT-116) cancer cell line by using the MTT [17,24]. The cells were incubated overnight at 37 °C in 5% CO_2_/air in microtiter plates. Tested compound, etoposide (positive control), and DMSO (negative control) were added to the top row of a 96-well microtiter plate and serially diluted (1:4) downward. After a 72 h incubation, cell viability was determined colorimetrically using a Molecular Devices Emax microplate reader (490 nm), recording the amount of MTS (3-(4,5-dimethylthiazol-2-yl)-5-(3-carboxymethoxy phenyl)-2-(4-sulfophenyl)-2*H*-tetrazolium) reduced to formazan using the Cell Titer 96 AQueous non radioactive cell proliferation protocol (Promega,Madison, WI, USA). Minimum inhibitory concentration (IC_50_, μM) values were calculated using the program SOFTmax PRO (Molecular Devices, Sunnyvale, CA, USA). The results were shown in Table 2.

### 3.7. Antifungal Assay with C. albicans

The minimal inhibitory concentration (MIC; lowest concentration of the compound able to inhibit microorganism growth) of compound **1** was evaluated against two strains of *Candida albicans* ATCC 32354 (wild type) and ATCC 90873 (amphotericin B-resistant). These strains were purchased from the American Type Culture Collection (ATCC). Inhibitory activity was determined by a standard microdilution liquid antifungal assay [25]. *Candida albicans* was incubated overnight at 37 °C in RPMI 1640 media (GibcoBRL, Invitrogen Corp, Carlsbad, CA, USA) and aliquots transferred to 96-well plates the next day. The indicator Alamar Blue was added to the *C. albicans* culture before they were transferred to the plates. Samples were added along with amphotericin B (Sigma, St. Louis, MO, USA) and DMSO (solvent) as positive and negative controls, respectively, and serially diluted. The plates were then incubated overnight for 14–16 h. Minimum inhibitory concentration (MIC) values were determined by the change in color from blue to pink of the media according to the indicator Alamar Blue. The results of the activity were shown in Table 2.

## 4. Conclusions

In conclusion, the investigation of the Red Sea sponge *Theonella swinhoei* led to isolation of a new bicyclic glycopeptide, theonellamide G (**1**). The structure was determined using extensive spectroscopic studies. Theonellamide G showed potent antifungal activity towards wild and amphotericin B-resistant strains of *Candida albicans* with IC_50_ of 4.49 and 2.0 μM, respectively, compared to 1.48 μM for the positive antifungal control amphotericin-B against the wild type. Additionally, it displayed cytotoxic activity against the human colon adenocarcinoma cell line (HCT-16) with IC_50_ of 6.0 μM, compared to 2.0 μM for etoposide (positive anticancer control). 

## Supplementary Files

Supplementary File 1 Supplementary Information (PDF, 1469 KB)
