# Supplementary material for: Theonellamide G, a Potent Antifungal and Cytotoxic Bicyclic Glycopeptide from the Red Sea Marine Sponge Theonella swinhoei
Source: Mar Drugs. 2014 Apr 1;12(4):1911–23. doi: 10.3390/md12041911 (PMC4012434; doi:10.3390/md12041911)
Supplement: Supplementary File 1 — Supplementary Information (PDF, 1469 KB) [file marinedrugs-12-01911-s001.pdf]

Supplementary Information

Figure S1. 600 MHz 1H NMR Spectrum of compound **1** (DMSO-d_6:H_2O, 4:1).
Figure S2. 150 MHz 13C NMR Spectrum of compound **1** (DMSO-d_6:H_2O, 4:1).
Figure S3. DQF COSY Spectrum of compound **1** (DMSO-d_6:H_2O, 4:1).
Figure S4. HSQC Spectrum of compound **1** (DMSO-d_6:H_2O, 4:1).
Figure S5. HMBC spectrum of compound **1** (DMSO-d_6:H_2O, 4:1).
Figure S6. NOESY spectrum of compound **1** (DMSO-d_6:H_2O, 4:1).

Figure S1. 600 MHz 1H NMR Spectrum of compound **1** (DMSO-d_6:H_2O, 4:1).

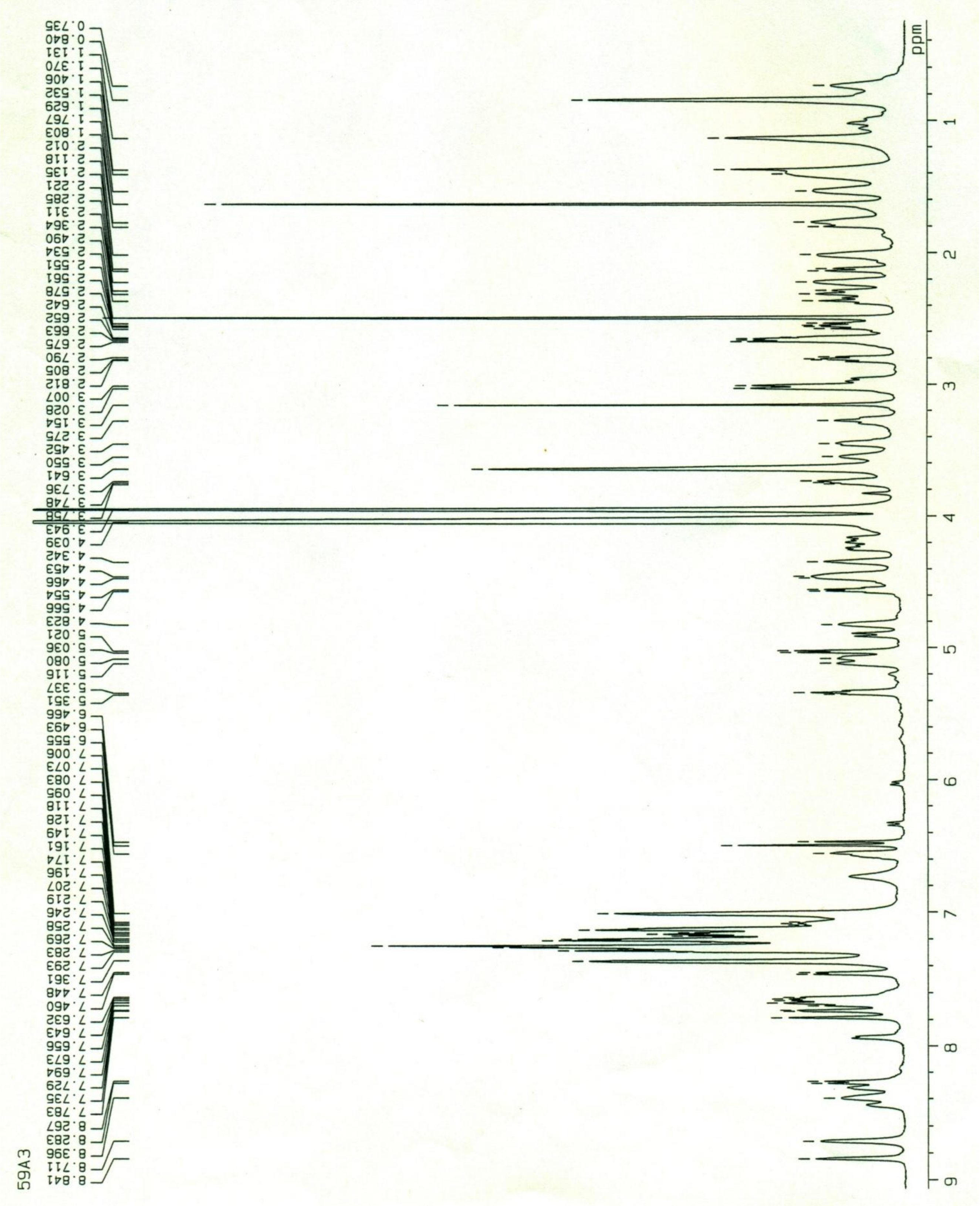

Figure S2. 150 MHz 13C NMR Spectrum of compound **1** (DMSO-d_6:H_2O, 4:1).

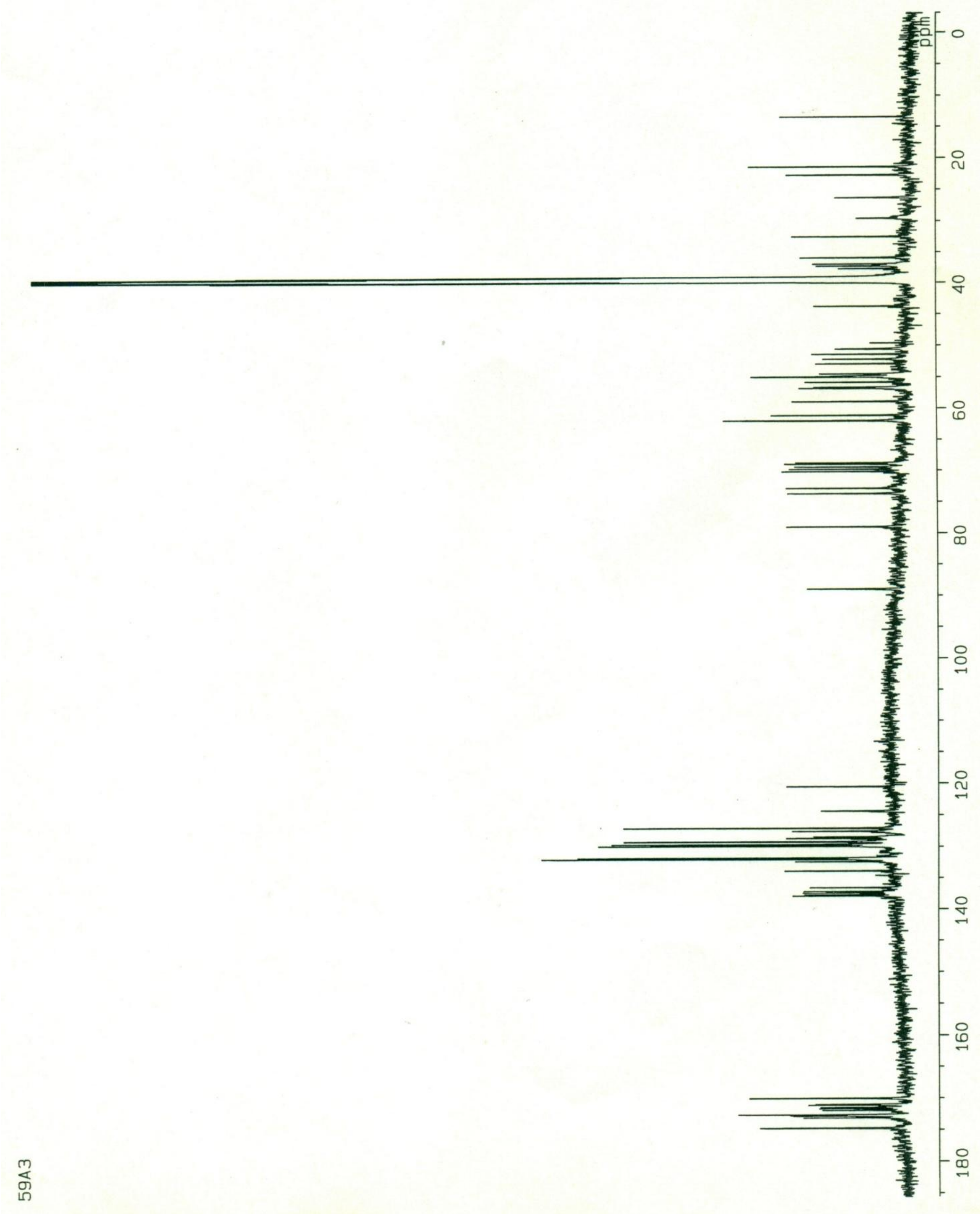

Figure S3. DQF COSY Spectrum of compound **1** (DMSO-d$_6$:H_2O, 4:1).

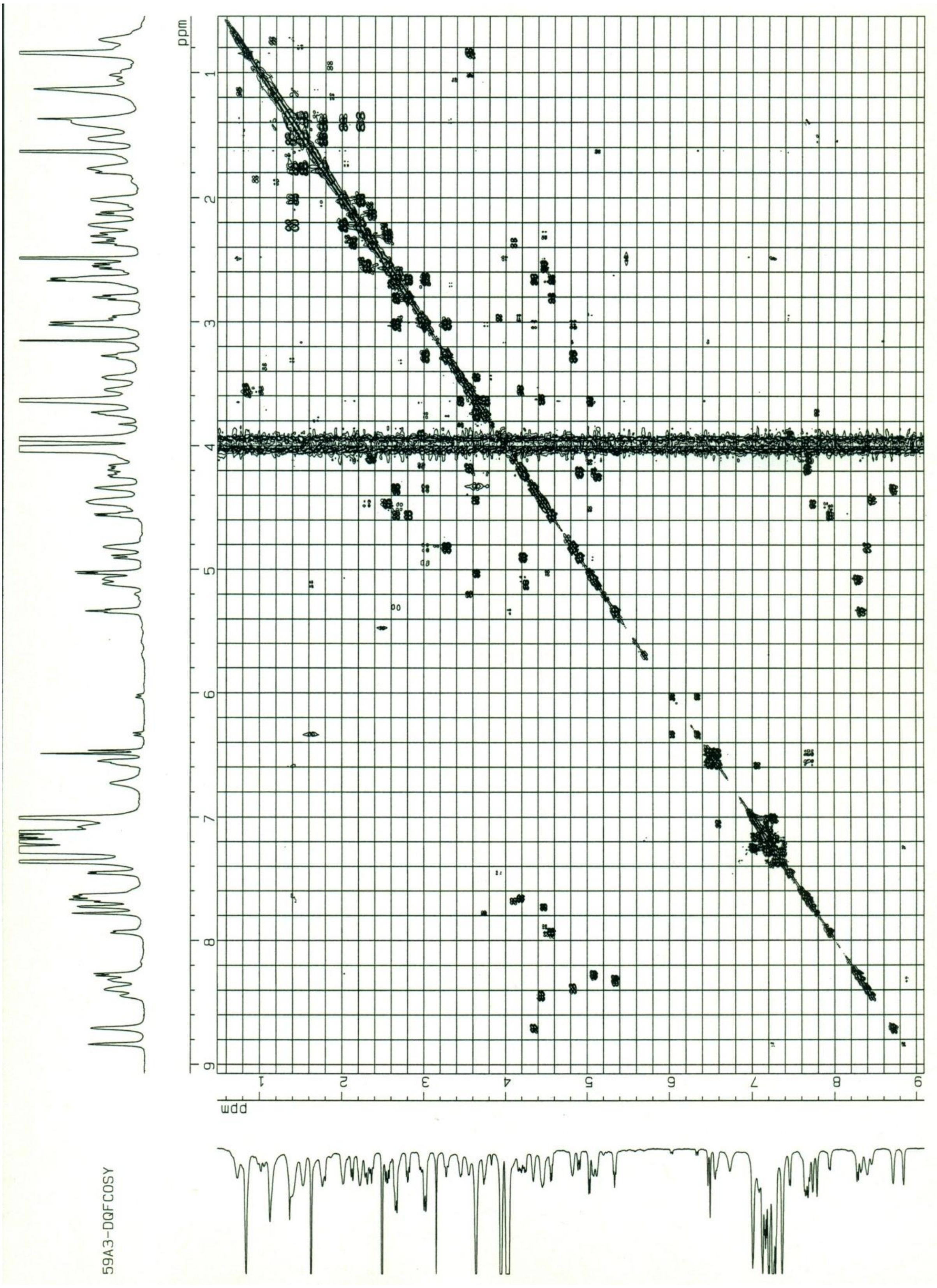

Figure S4. HSQC Spectrum of compound **1** (DMSO-d_6:H_2O, 4:1).

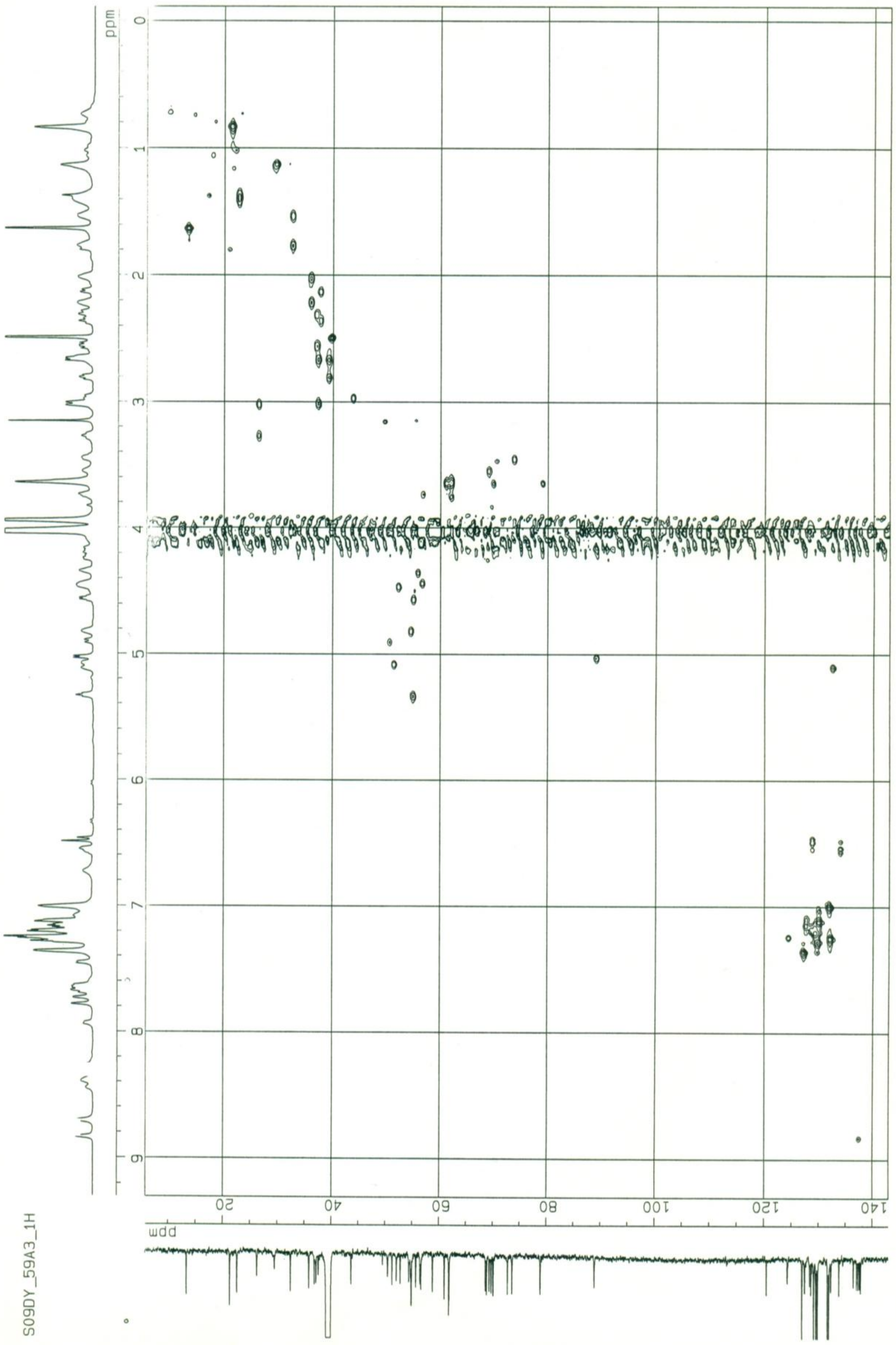

Figure S5. HMBC spectrum of compound **1** (DMSO-d_6:H_2O, 4:1).

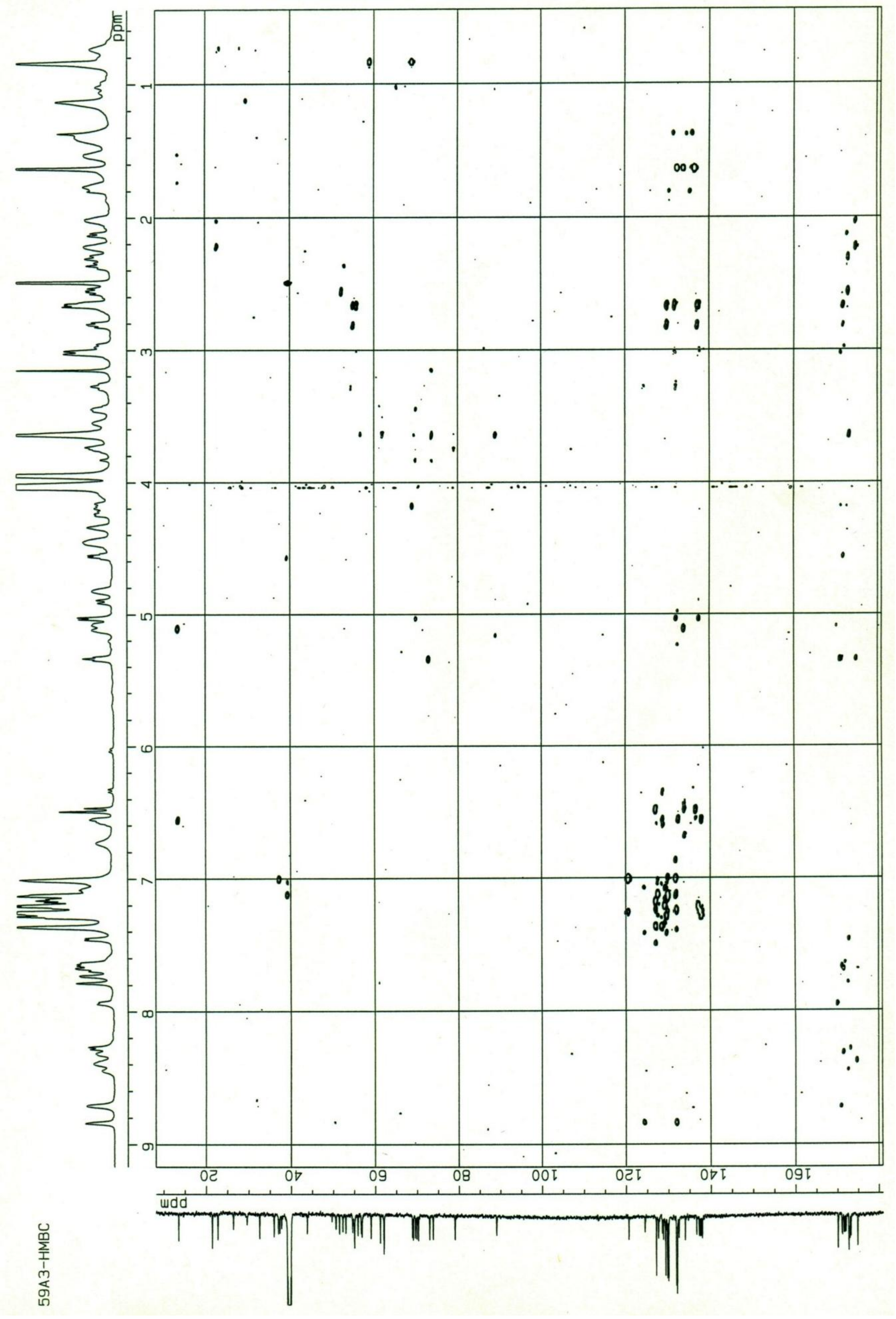

Figure S6. NOESY spectrum of compound **1** (DMSO-d$_6$:H_2O, 4:1).

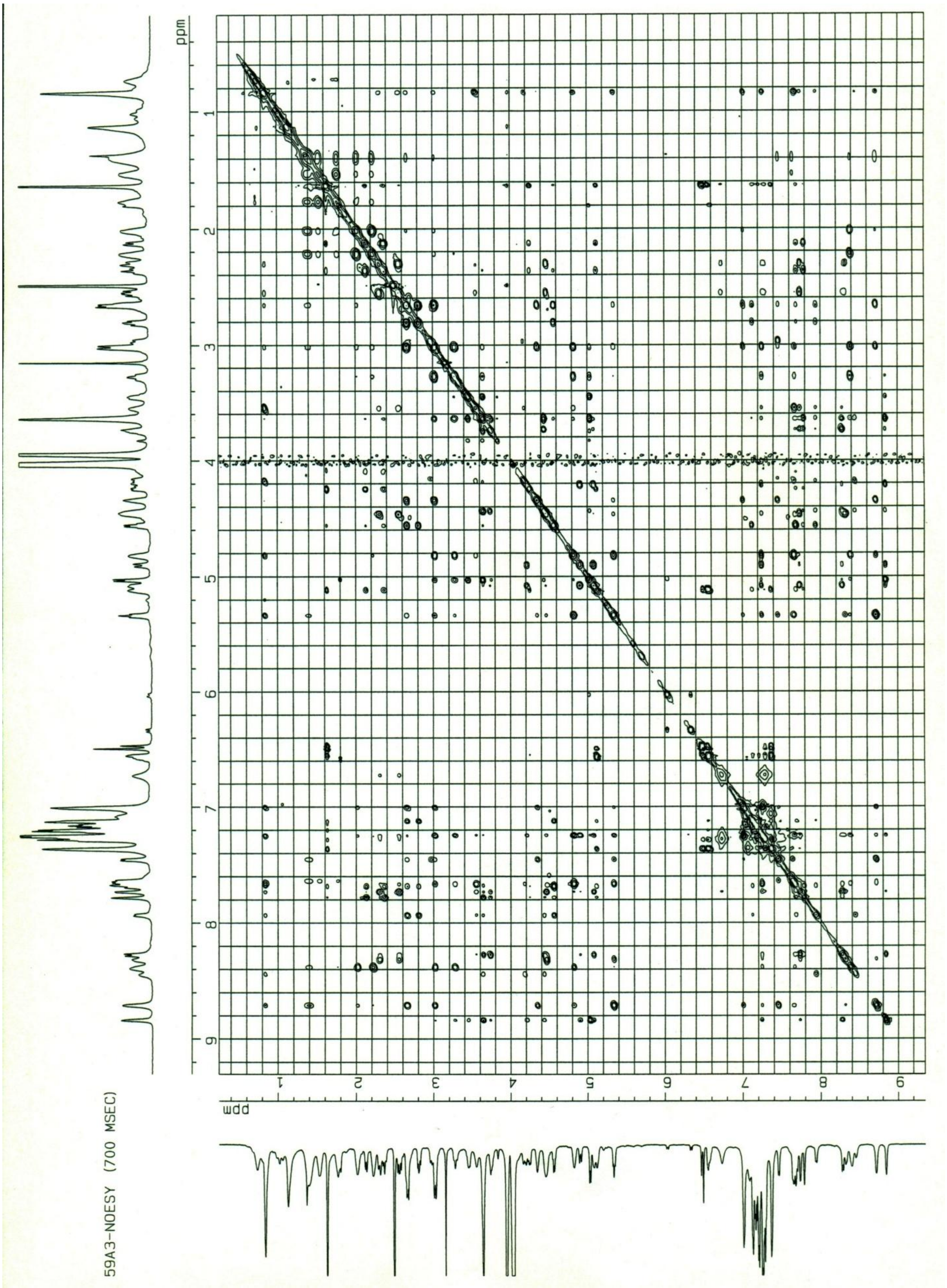

© 2014 by the authors; licensee MDPI, Basel, Switzerland. This article is an open access article distributed under the terms and conditions of the Creative Commons Attribution license (http://creativecommons.org/licenses/by/3.0/).
